# Non-invasive evaluation of neurovascular coupling in the murine retina by dynamic retinal vessel analysis

**DOI:** 10.1371/journal.pone.0204689

**Published:** 2018-10-04

**Authors:** Walid Albanna, Konstantin Kotliar, Jan Niklas Lüke, Serdar Alpdogan, Catharina Conzen, Ute Lindauer, Hans Clusmann, Jürgen Hescheler, Walthard Vilser, Toni Schneider, Gerrit Alexander Schubert

**Affiliations:** 1 Institute for Neurophysiology, University of Cologne, Cologne, Germany; 2 Department of Neurosurgery, RWTH Aachen University, Aachen, Germany; 3 Department of Medical Engineering and Technomathematics, FH Aachen University of Applied Sciences, Aachen, Germany; 4 Tranlational Neurosurgery and Neurobiology, RWTH Aachen University, Aachen, Germany; 5 IMEDOS Systems UG, Jena, Germany; University of Florida, UNITED STATES

## Abstract

**Background:**

Impairment of neurovascular coupling (NVC) was recently reported in the context of subarachnoid hemorrhage and may correlate with disease severity and outcome. However, previous techniques to evaluate NVC required invasive procedures. Retinal vessels may represent an alternative option for non-invasive assessment of NVC.

**Methods:**

A prototype of an adapted retinal vessel analyzer was used to assess retinal vessel diameter in mice. Dynamic vessel analysis (DVA) included an application of monochromatic flicker light impulses in predefined frequencies for evaluating NVC. All retinae were harvested after DVA and electroretinograms were performed.

**Results:**

A total of 104 retinal scans were conducted in 21 male mice (90 scans). Quantitative arterial recordings were feasible only in a minority of animals, showing an emphasized reaction to flicker light impulses (8 mice; 14 scans). A characteristic venous response to flicker light, however, could observed in the majority of animals. Repeated measurements resulted in a significant decrease of baseline venous diameter (7 mice; 7 scans, p < 0.05). Ex-vivo electroretinograms, performed after in-vivo DVA, demonstrated a significant reduction of transretinal signaling in animals with repeated DVA (n = 6, p < 0.001).

**Conclusions:**

To the best of our knowledge, this is the first non-invasive study assessing murine retinal vessel response to flicker light with characteristic changes in NVC. The imaging system can be used for basic research and enables the investigation of retinal vessel dimension and function in control mice and genetically modified animals.

## Introduction

Neurovascular coupling (NVC) enables a transient increase in neural activity by increasing cerebral blood flow and metabolism [1]. During synaptic activity and activation of neurons, metabolic processes are stimulated for ATP production to fuel the activities of ion pumps. Metabolic substrates (glucose, oxygen) are assured through adjacent capillaries. In response to transient neural activity, nearby vessels dilate, vessel resistance is reduced and blood flow increases [2]. The exact mechanism was initially thought to be driven by lack of oxygen [3], but this concept is now discarded in favor of more complex mechanisms involving distinct vasoactive agents [4,5]. Astrocytes may participate in NVC by detecting an increase in neurotransmission, releasing those vasoactive agents from perivascular endfeet surrounding parenchymal arterioles [6]. Modulation of vessel diameter by oxygen and NO are described [7], but vast aspects of the reaction pathways involved remain obscure. The retina as an embryological part of the central nervous system may be suitable to study neurovascular principles of the brain by investigating retinal reactivity to locally enhanced neuronal activity non-invasively (“retina as a window to the brain”). In selected systemic diseases such as arterial hypertension [8] or diabetes mellitus [9,10], impairment of retinal NVC in response to light stimulation has been described, presumably due to characteristic functional and structural vascular abnormalities. As a possible clinical analogue, altered autoregulation in the brain was also observed after aneurysmal subarachnoid hemorrhage (SAH) contributing to disturbances of cerebral blood flow and resulting in poor clinical outcome [11–13].

Preliminary data on retinal vessel reactivity in patients after SAH using Dynamic Vessel Analyzer (DVA, IMEDOS Systems UG, Jena, German) suggest a characteristic role of NVC in neurological diseases [14,15]. Although NVC manifestation of retinal vessel response to flicker light in humans has been documented before [16,17], the underlying pathophysiology requires further clarification using basic research. So far, DVA has not been performed successfully in smaller rodents such as mice, which would allow for genetic modification, as well as characterization of vessel diameter and reactivity (NVC) in the context of available disease models.

Using a prototype of an adapted DVA, this study assesses the feasibility of retinal vessel analysis in a small rodent model for the first time.

## Materials and methods

### Animals

All experiments were performed using male mice (C57Bl/6 x 129SvJ). The mice (16–40 weeks old, 25–31 g) were housed at a constant temperature (20°C—22°C) in Makrolon type II cages, with illumination from 7 a.m. to 7 p.m. (light intensity at the surface of the animal cages was 5–10 lux) and access to food and water was ad libitum. The institutional and governmental committees on animal care approved the animal experimentation described in the text (Landesamt für Natur, Umwelt und Verbraucherschutz (LANUV) Nordrhein–Westfalen, Recklinghausen, Germany; 84–02.04.2016.A4555) which was conducted following accepted standards of humane animal care.

### RCrodent vessels analysis

**Fig 1:**
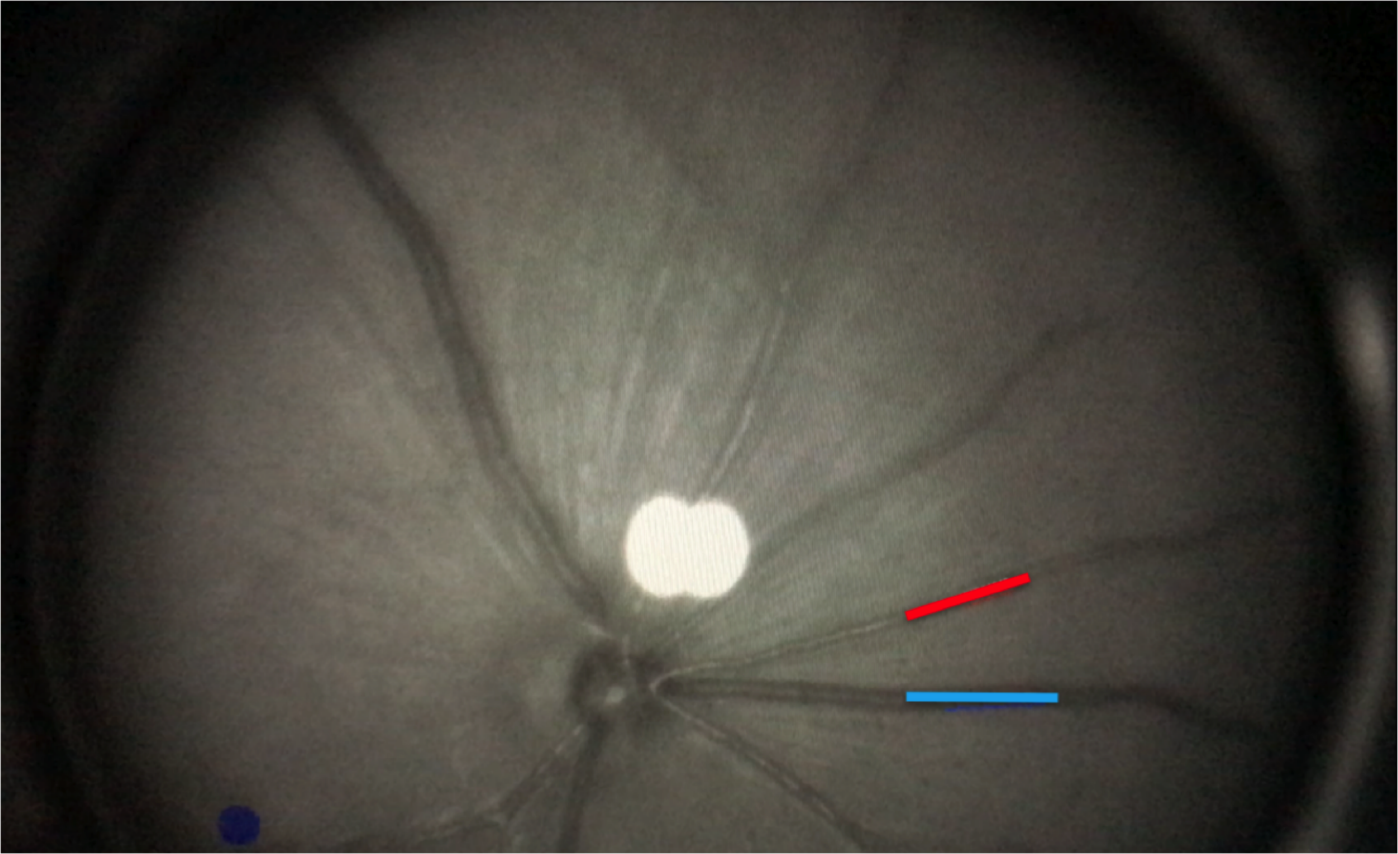
Measurement with DVA (RCrodent, IMEDOS Systems UG, Jena, Germany). Murine retina as assessed with DVA. Arterial (red) and venous (blue) segments are marked.

A prototype of the adapted Dynamic Vessel Analyzer (DVA) was used (RCrodent, IMEDOS Systems UG, Jena, Germany). The original device enables dynamic vessel analysis as a function of time by applying flicker light impulses at defined frequencies (12.5 Hz, 20 s, 530 nm) as reported in rats [18]. The light intensity was set at 30 lux. The mice were dark-adapted overnight (12 hours), and the left eye was dilated via application of a mydriatic agent (Tropicamide, Mydriaticum Stulln UD, Pharma Stulln GmbH, Stulln, Germany). A dedicated mouse contact lens with the following specifications was used to protect the cornea: polymethylmethacrylate, Back Optic Zone Radius 1.7 mm, diameter 3.2 mm, zero dioptric (Cantor & Nissel, Brackley, UK). Anesthesia was induced via intraperitoneal injection of ketamine hydrochloride (66.7 mg/kg body weight; Ketanest, Parke-Davis/Pfizer, Berlin, Germany) and xylazine hydrochloride (6.7 mg/kg body weight, Rompun 2% Bayer Vital, Leverkusen, Germany). All mice were positioned on a heating plate to maintain a constant body temperature of 37°C (**Fig 1**).

**Fig 2:**
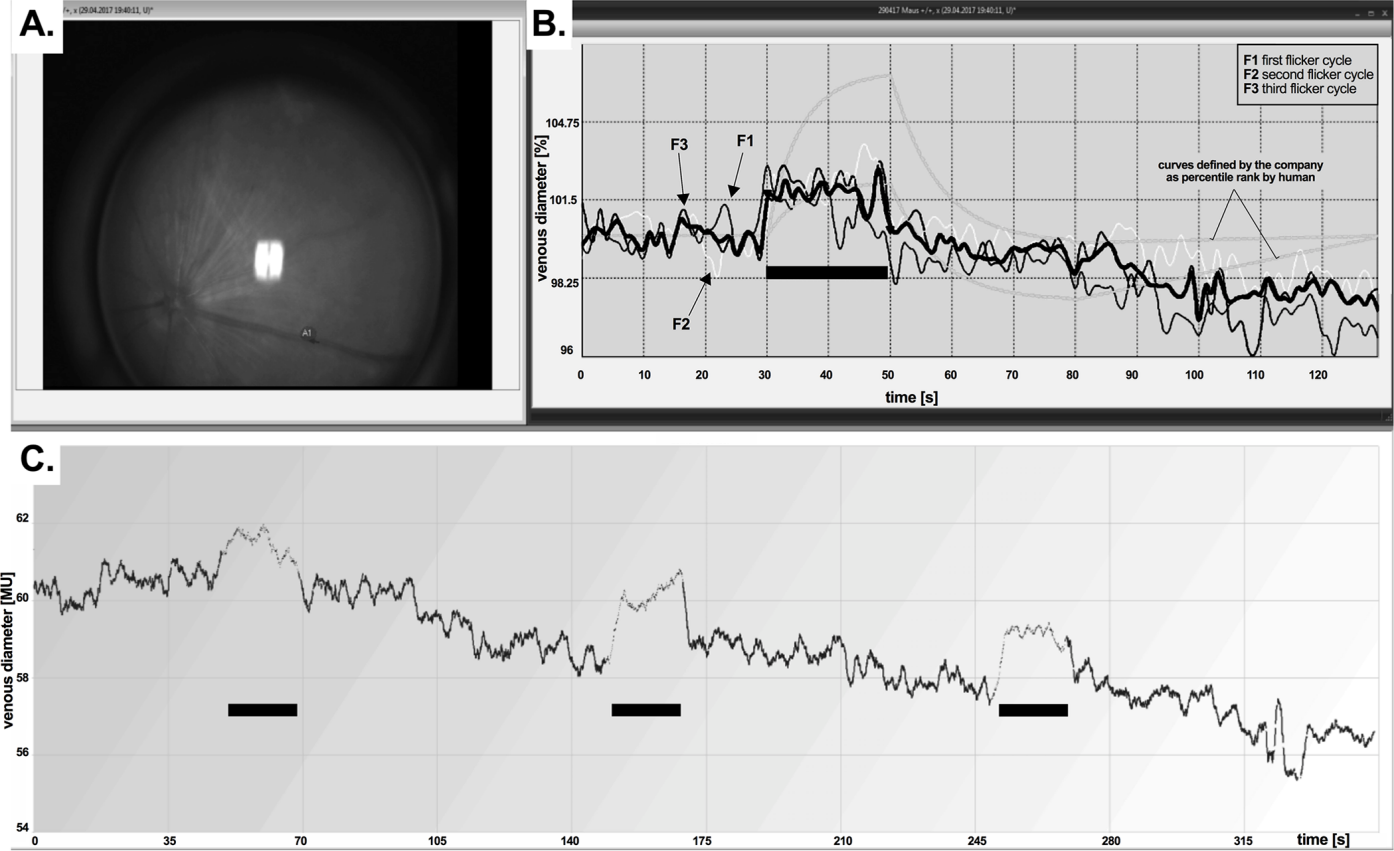
An example of DVA measurement in a mouse and primary data evaluation. **A.** Diameter of retinal venous segment was assessed. **B.** Retinal venous response is averaged from the 3 cycles (thin lines; F1: first flicker cycle, F2: second flicker cycle, F3: third flicker cycle) and plotted as relative vessel diameter change (mean value, thick black line) over time in % to the baseline. C. Absolute vessel diameter measured in MU is plotted over time during 350 s of the assessment. Three cycles of flicker responses (black horizontal bars) with characteristic vessel dilation phases are shown.

We followed the standard 350 s measurement protocol by IMEDOS Systems for human studies [8]. After baseline assessment of 50 s, monochromatic rectangular flicker stimulation (530, 12.5 Hz, 20 s) was applied for three consecutive cycles (**Fig 2**). In addition to the automated analysis performed by the commercial DVA software, further parameters of dynamic vascular response were derived and analyzed as described previously [19] by a template-set with corresponding macros (Microsoft Office Excel 2016, Californian, USA) to filter, process, and analyze numerical data from the original DVA-assessment [20,21]. Briefly, absolute vessel diameter and distances within the retina were gauged by arbitrary measuring units (MU) where 1 MU is approximately 1 μm in the mouse eye. The conversions from the imaging system were calculated based on the size of a theoretical standard eye. To compare flicker responses in different animals, relative change in vessel diameter was calculated. All retinae underwent three cycles of flicker application, and the corresponding three individual response curves (30 s of baseline before flicker application, 20 s during flicker application and 80 s after flicker application) were averaged. To reduce the high-frequency noise of temporal vessel diameter changes the time courses were averaged using running median with a window of 4 seconds and the corresponding back shift. The following parameters were calculated:

- mean maximal dilation in response to flicker (% to baseline)
- dilation at flicker cessation (% to baseline)
- time until maximal dilation (s)
- mean maximal constriction, (% to baseline)
- reactive magnitude: difference between mean maximal dilation and mean maximal constriction (% to baseline).
- AUC during flicker (%*s): area under the curve during 20 s of the flicker stimulation.
- time of center of gravity at flicker (s): time to reach the “center of gravity” of the area under the curve over the baseline between flicker initiation and the first baseline intersection after the peak dilation [21].

Averaged for a group, time courses of vessel diameter changes were created following the technique described previously [19,22]. Briefly, for each time point of the ensuing average curve, a median of all individual relative vessel diameters in the group at this time point was plotted.

To assess the effect of repeated DVA analyses, selected mice underwent repeated DVA measurements, alternating with interposed recovery periods with dark adaption (mean 11.0 (9.0–12.5) min. To explore NVC in retinal vessels in different areas of the murine fundus, measurements were recorded digitally and analyzed off-line for additional vessel segments. The quality of DVA recordings was assessed semi-objectively using the cumulative scoring method reported previously [21]. The score ranged from 0 (“inadequate”) to 5 (“excellent quality”). Only DVA recordings with score values ≥ 2.0 were included for this evaluation. After the DVA procedure, the ERGs were measured post-mortem (ex vivo).

### Retina isolation and ERG-recordings

After the DVA procedure, ex vivo ERGs were performed after dark adaptation (minimum of 10 mins between last DVA and retina isolation). Mice were sacrificed by cervical dislocation under a dim red light, and the eyes were extirpated immediately. Enucleated eyes were protected from light and transferred into carbogen-saturated (95% O_2_ / 5% CO_2_) Ames medium and into the recording chamber as described previously [23]. From the dark-adapted retina, electroretinograms in response to a single white light flash (500 ms) were recorded at intervals of 3 min at 27.5°C and with a constant superfusion of nutrient solution at 2 ml/min. The flash intensity was set to 63 mlux at the retinal surface using calibrated neutral density filters. The ERGs were amplified using ADIntruments (PowerLab 8/35, Animal Bio Amp FE136, ADIntruments, Oxford, UK). For quantification of transretinal signaling, we calculated b-wave amplitudes and their implicit time. The b-wave amplitude was measured from the trough of the a-wave to the peak of the b-wave [23]. For each experiment, a new murine retina was transferred to the recording chamber.

### Statistical analysis

Metric and ordinal scaled data are shown as median [1^st^ quartile– 3^rd^ quartile]. If normally distributed, the metric scaled data is presented as mean ± standard deviation (SD) instead. For categorical variables, data are presented as numbers and percentage. Two-sided Student t-test was used for the comparison of quantitative parameters in case of normal distribution. If applicable, Wilcoxon test or Mann-Whitney-U-test were used instead. In this study, statistical comparisons were carried out exploratively without correction for multiple comparisons to show tendencies and stronger effects of retinal venous response. Statistical significance was set at p < 0.05, statistical results with p < 0.1 were accepted as trend. All analyses and data presentation were performed with Excel (Microsoft Office Excel 2016, Californian, USA), SPSS v. 21 (IBM Chicago, Illinois, USA) and GraphPad Software (GraphPad Prism, Inc, La Jolla, USA).

## Results

### Venous response in DVA

**Fig 3:**
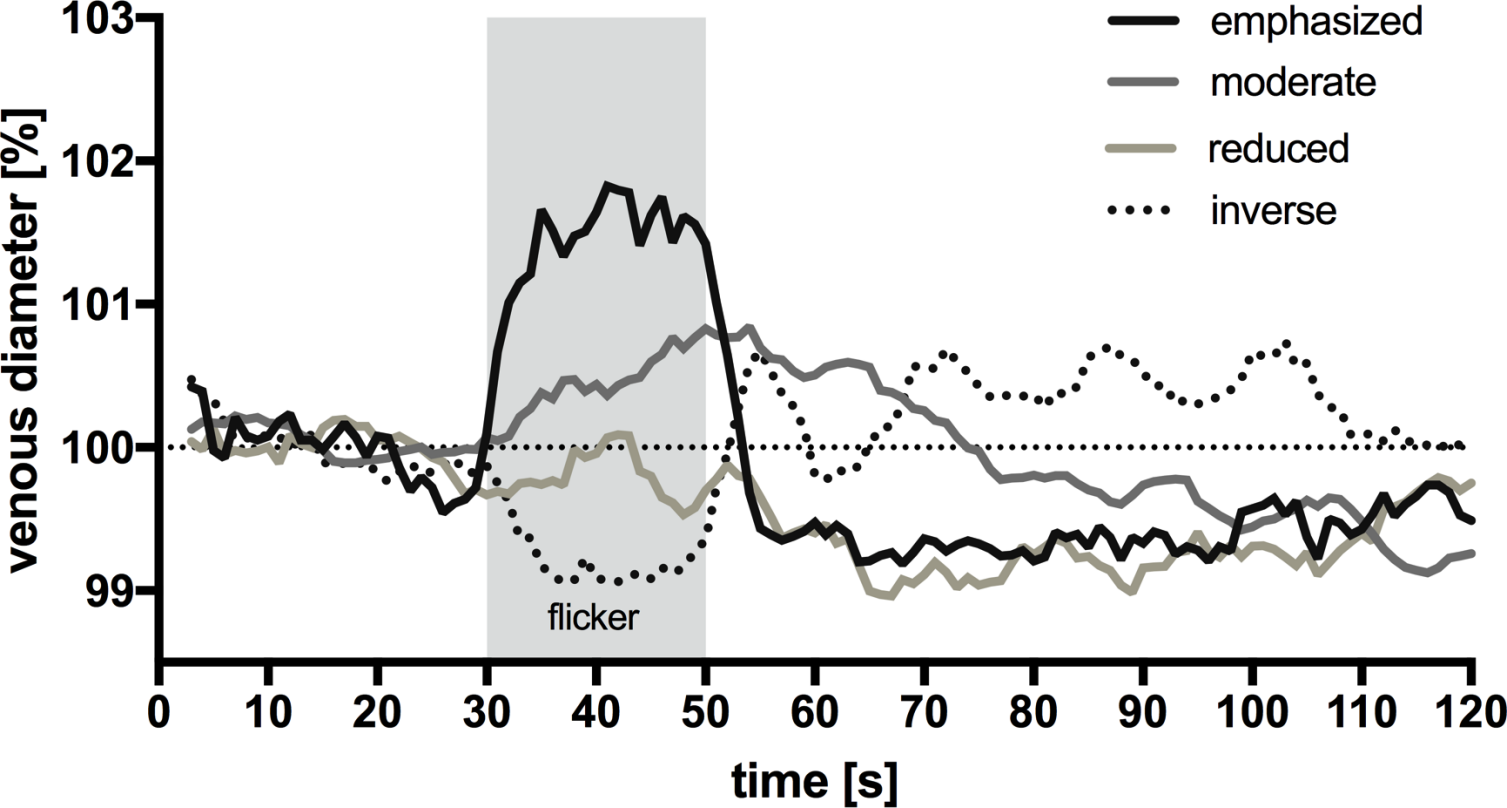
Venous reaction by category. Total 90 measurements in 21 mice (explanation in text). For each cohort, recordings are shown to demonstrate the effect of flicker stimulation on the venous diameter. Emphasized reaction (n = 17 / 90), moderate reaction (n = 33 / 90), reduced visible reaction (n = 20 / 90) and inverse reaction (n = 7 / 90).

In all 21 mice, we were able to observe venous reaction to flicker in at least one of the measured vessel segments. However, not all the venous segments of a mouse reacted to the same extent. We classified the measured retinal venous reactions as follows (**Fig 3**):

- **emphasized** reaction with maximal vessel dilation ≥ 1.5%, n = 17 / 90 → 19%.
- **moderate** reaction with maximal vessel dilation ≥ 0.5% and < 1.5%, n = 33 / 90 → 37%.
- **reduced**, but discernible reaction < 0.5%, n = 20 / 90 → 22%.
- no reaction, n = 13 (14%).
- **inverse** reaction (constriction) as a response to flicker application: n = 7 / 90 → 8%.
- Vasodilation (reduced, moderate or emphasized) was observed in 70 out of 90 measurements (78%).

To characterize an optimal retinal venous response to flicker, we compared the best (emphasized) to the worst vessel reactions in each mouse, and maximal vessel reaction in response to flicker was considered a key parameter. The results are represented in **S4 Fig** and on **S1 Table**. Murine retinal venous response to flickering light was smaller compared to the retinal venous response in healthy humans [20,24] (**S4 Fig**).

### Vessel responses during repetitive flicker cycle (single DVA)

To assess the impact of repetitive stimulation with flicker, 21 venous measurements with the best imaging quality from 21 animals were analyzed. The response to the first, second and third cycle was compared intra-individually. Upon completion of three cycles, 14 animals showed a continuous decrease in vessel diameter (**S5 Fig**), while in the remaining seven animals a continuous increase in vessel diameter was observed (**S5 Fig**). However, when averaged for all animals, the change in diameter was not altered significantly from cycle to cycle (**S2 Table**). In animals with continuous decrease, mean maximal dilation was significantly lower than in animals with a continuous increase in diameter: 1.3 (0.7–1.8) % *vs*. 2.3 (1.2–3.1) % (p = 0.038). However, none of the other parameters showed significant differences.

When comparing animals dichotomized according to the change in diameter, animals with decreasing diameter (n = 14) showed a more emphasized dilation in response to flicker at each cycle than animals with a continuous increase in diameter (n = 7). Maximal venous dilation calculated as the maximal value within the corresponding cycle amounted to:

*Cycle 1*: 0.7 (-0.6 − 1.4) % *vs*. 1.5 (-0.3 − 2.9) %, p = 0.40;

*Cycle 2*: 0.8 (0.0 − 1.8) % *vs*. 3.4 (1.9 − 4.9) %, p = 0.038;

*Cycle 3*: 0.9 (0.4 − 2.8) % *vs.* 1.7 (-0.8 − 4.3) %, p = 0.75.

### Effect of repeated measurements (multiple DVA) on neuronal vessel interactions

In order to investigate the effect of repeated measurement, we compared seven measurements of seven mice in the same retinal venous segment during primary and repeated RVA-assessments (**Table 1, S6 Fig**). Average time interval between measurements was 11.0 (9.0–12.5) min. Absolute baseline vessel diameter of repeated measurements was significantly smaller than the baseline of the primary measurement (p < 0.05). On average, venous reaction of a repeated measurement was reduced and delayed, and however, the differences did not reach statistical significance between subsequent measurements (p = 0.106).

**Table 1:** Parameter of retinal venous reaction to flickering light: primary vs. repeated measurement. n = 7, age 4.6 (4.1–10.0) mo.; median (1^st^ quartile– 3^rd^ quartile), significance with Wilcoxon test. Explorative testing without correction to multiple comparisons.

parameter/group	primary measurement	Repeated measurement	p—value
data quality, [subjective score 1.0–5.0 ]	4.0 (3.5–4.5)	4.0 (4.0–4.5)	0.874
venous diameter, [MU]	61.1 (60.6–63.0)	58.6 (51.3–60.5)	**0.028**
mean maximal venous dilation, [% baseline]	0.9 (0.6–1.7)	0.5 (0.2–0.8)	0.271
time of maximal venous dilation, [s]	12.0 (9.5–14.0)	16.0 (6.5–26.0)	0.612
venous dilation at the flicker cessation, [% baseline]	0.8 (0.2–1.2)	0.0 (-0.1–0.6)	0.235
venous reactive magnitude, [% baseline]	2.5 (1.7–2.6)	1.3 (1.1–1.6)	0.106
venous AUC during the flicker, [%*s]	14.4 (3.6–26.9)	1.2 (-1.2–9.9)	**0.091**
venous time of center of gravity at flicker, [s]	16.6 (11.4–38.6)	27.4 (15.7–47.8)	0.345
mean maximal venous constriction, [% baseline]	-0.9(-2.3 –-0.3)	-0.6 (-1.4 –-0.2)	0.456

### ERG and the effect of illumination of DVA on the retina

**Fig 4:**
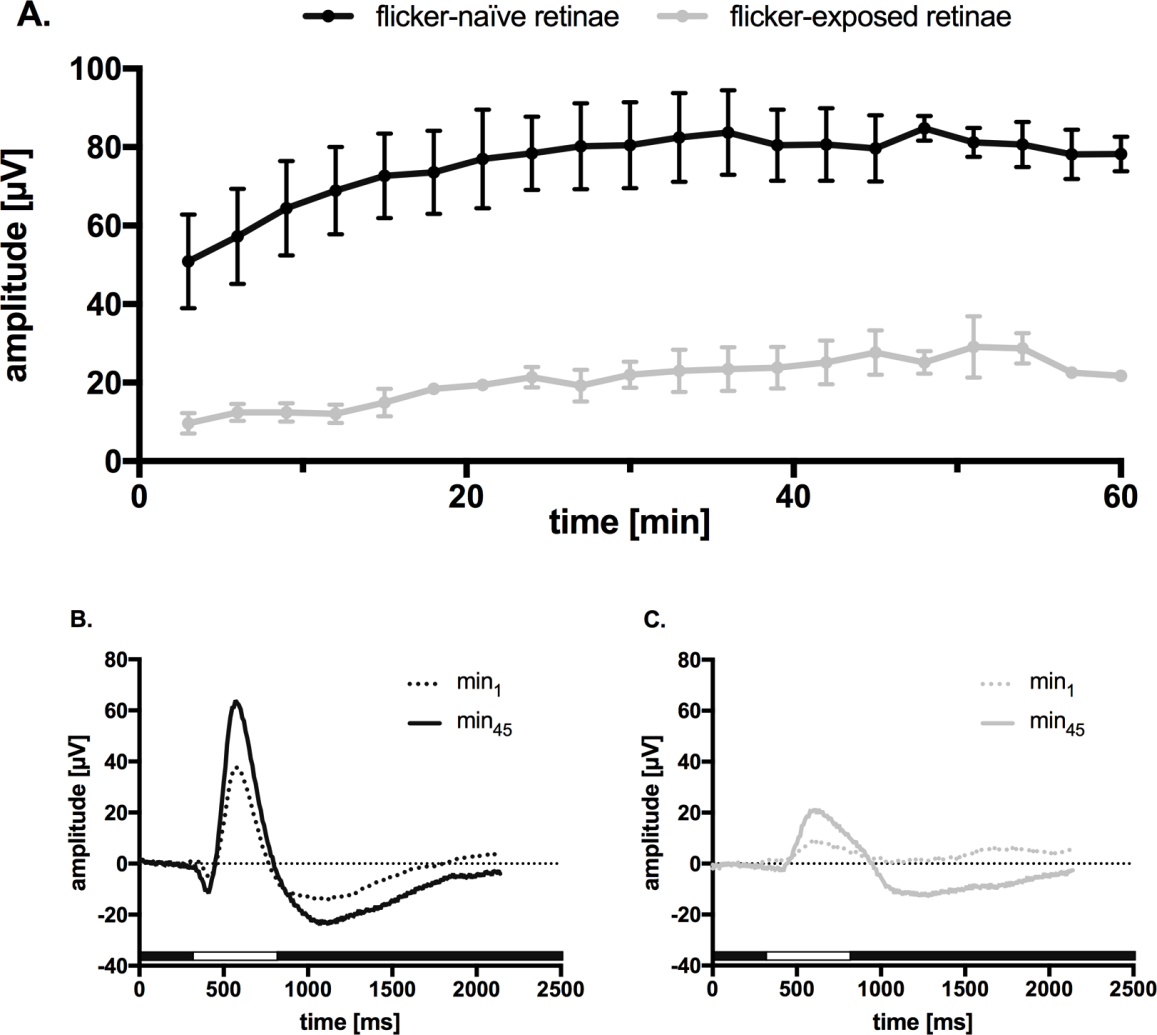
Electroretinographic (ERG) responses of the isolated and superfused murine retina. **A.** Repetitive light stimulation (63 mlux) every 3 min. The b-wave amplitude increased during the initial equilibration period of about 60 min. Flicker-naïve retinae are shown in black (n = 4), retinae undergoing DVA (flicker-exposed) are demonstrated in gray (only three out of four retinae showed ERG responses). The nutrient solution was superfused at the same basal speed (2 ml / min). **B.** Corresponding ERG traces from data of four flicker-naïve retinae is presented in panel **A**; after 1 min superfusion (51 ± 23μV, dotted black curve) and 45 min (80 ± 17 μV, black curve). **C.** Superimposed ERG traces from the data of retinae underlying DVA and presented in panel A. 1 min superfusion (10 ± 4 μV, dotted gray curve), 45 min (28 ± 10 μV, gray curve). The duration of light stimulus was always 500 ms, and it is indicated by a white bar below the traces.

To study the effect of DVA-illumination on the retina (30 lux over 350 sec), electroretinograms (ERGs) of both eyes were performed. ERGs, recorded in response to repetitive stimulation (10 min) of the retina are shown in **Fig 4**. B-wave amplitudes were reduced in exposed retinae compared to the non-exposed retinae at the beginning (min_1_: 10 ± 4 μV vs. 51 ± 23 μV, p = 0.034), and after equilibration (min_45_: 28 ± 10 μV vs. 80 ± 17 μV, p = 0.005). Implicit times were extended in exposed retinae at the beginning (min_1_: 317 ± 72 ms vs. 243 ± 13 ms, p = 0.093) and after equilibration (min_45_: 300 ± 0 ms vs 243 ± 13 ms, p = 0.0006). One retina was nonresponsive after DVA procedure.

### Size- and location-oriented vessel analyses of the murine retina

Based on previous clinical data, we hypothesized that smaller murine retinal veins show more emphasized relative vessel diameter change in response to flicker than larger vessels. 90 measurements were arranged in ascending order of measured retinal venous diameter values. We compared 30 measurements with lower baseline diameter (mean 43.5 MU: 37.2–46.2 MU) and 30 with higher baseline diameter (mean 63.8 MU: 61.7–65.6 MU) (**S3 Fig, Table 2**). Smaller veins tended to respond to flickering light with less dilation and constricted more after flicker cessation than vessels with larger diameters (p < 0.05). Retinal venous reaction proximal to the optic nerve head (ONH) and distal to the ONH segments were also compared. Vessel segments located within 1.5 ONH-diameter away from ONH rim were classified as proximal, while segments more than 4 ONH diameters distant were categorized as distal. Venous diameter was comparable in both subgroups. Distance to ONH did not have any significant impact on the analyzed flicker parameters in our study (**S3 Fig, S2 Table**).

**Table 2:** Parameter of retinal venous reaction to flickering light: small vs. large vessel diameter. n = 30; age: 4.7(4.0–5.7) Mo. vs. 4.6 (4.2–5.4) Mo. (p = 0.829); median (1^st^ quartile– 3^rd^ quartile), significance with Mann-Whitney-U-test. Explorative testing without correction to multiple comparisons.

parameter/group	small vessel diameter	large vessel diameter	p—value
data quality, [subjective score 1.0–5.0 ]	4.0 (3.5–4.5)	4.0 (4.0–4.5)	0.874
venous diameter, [MU]	43.5 (37.2–46.2)	63.8 (61.7–65.6)	**<0.001**
mean maximal venous dilation, [% baseline]	0.7 (0.2–1.1)	0.9 (0.4–1.6)	0.152
time of maximal venous dilation, [s]	18.0 (12.0–24.8)	18.0 (10.3–23.8)	0.740
venous dilation at the flicker cessation, [% baseline]	-0.1 (-0.6–0.7)	0.5 (0.0–0.8)	**0.039**
venous reactive magnitude, [% baseline]	1.8 (1.3–2.4)	1.5 (1.1–2.4)	0.289
venous AUC during the flicker, [%*s]	0.4 (-9.1–10.2)	4.1 (0.8–9.9)	**0.066**
venous time of center of gravity at flicker, [s]	18.8 (12.3–27.5)	21.7 (14.2–40.6)	0.546
mean maximal venous constriction, [% baseline]	-1.5 (-1.7 –-0.6)	-0.8 (-1.1 –-0.5)	**0.019**

### Arterial response in DVA

Assessment of murine retinal arteries with DVA was technically feasible only in 8 animals (14 measurements). In this subgroup, an emphasized retinal arterial reaction was observed. Herein, the average response featured a similar structural shape to the retinal arterial response in humans consisting of a prompt dilation to 1.3 (1.0–1.7) % after the beginning of stimulation (mean maximal dilation) and the ensuing arterial constriction thereafter to -1.6 (-2.6 –-0.9) % (mean maximal constriction). An average summarized arterial reaction is shown in **S2 Fig**.

However, in the majority of measurements, no robust, reliable arterial reaction to flicker was detected due to anatomical constraints such as light reflection and faulty automated segmentation.

## Discussion

Neuronal activity triggers a demand-driven vasomotor response (NVC), and the underlying mechanism of NVC has gained more interest in recent years. Techniques to assess the integrity of NVC, however, are often invasive. Using the retina and its vascular unit may offer distinct advantages because of their shared embryological origin with the brain. The retina is readily assessable and can be examined non-invasively. Changes of NVC in the retina ascertainable in human beings has already been correlated with progression in a variety of systemic diseases, including arterial hypertension [8] and diabetes mellitus [9,10] or neuronal diseases such as stroke [25] and subarachnoid hemorrhage [14]. After subarachnoid hemorrhage, cerebral calcium signaling is altered, changing the polarity of NVC response in the brain from vasodilation to vasoconstriction [26–28]. Early detection of impaired NVC is indispensable to anticipate neuronal functional deterioration as well as microcirculatory damage, but fundamental assessment is needed to improve our understanding of the underlying pathophysiology. In view of a potential translation into a clinical context, a non-invasive technique to assess NVC would be preferable. However, successful non-contact retinal imaging to study NVC in genetically modifiable rodents, such as mice, has not been yet reported.

### Reliability of retinal findings

In this project, we present an imaging system using retinal imaging for dynamic retinal vessel analysis in the mouse to evaluate vessel diameter changes in response to NVC. Stable readings for retinal veins were obtained, but are suboptimal for arteries. Because of low contrast and a mirroring effect, the current version of RCrodent DVA does not yet allow for reproducible arterial measurements in all mice and is a clear limitation to this study. However, we were able to assess accentuated retinal arterial response in 14 arterial segments of 8 mice (**S2 Fig**).

Reproducible, stable results were obtained for the venous reaction, based on a superior contrast and therefore a better signal-noise ratio for video analysis and automated segmentation. Imaging qualities were comparable between the subgroups (see Tables) so that the extent of retinal vessel changes were unaffected in our analyses. In our cohort, we were able to measure a characteristic pattern of NVC in the retina regarding vasodilation and, in some cases, its inversion to vasoconstriction; these findings are in agreement with previous findings in the isolated retina of the rat [29].

The validity of the murine DVA measurements was investigated in different ways by classifying the 90 responses recorded from 21 mice. Although venous reactions were considerably smaller compared to human measurements (**S4 Fig**), a consistent interplay between flicker stimulation and changes in vessel diameter shows that dynamic retinal vessel analysis in murine eyes is feasible. One possibility for the smaller effect of flicker in mouse than human is that mice were anesthetized, which may depress the neuronal responses that lead to increases in diameter, or alter complicate neurovascular coupling responses by adding additional factors that directly affect the vasculature.

In line with previous investigations on rats [30], this study showed comparable vein responses to flicker light at approximately 1–2%. Based on experiments on optimal flicker frequency range to evoke maximal retinal vessel response in humans [16] and the physiological correlation of this optimal frequency range with flicker fusion threshold, we assume higher retinal vessel reactions in mice at higher flicker frequencies; those alterations, however, will require further modification of the existing hardware. Whether parameters of flicker stimulus including frequency, duration intensity and the shape of the illumination course would influence murine retinal vascular response will require further investigation.

### Differences in retinal vascular responses

We observed both an increase and a decrease in retinal venous dimension during a recording protocol of 350 s (**S5 Fig**). Interestingly, animals with an increase have shown a more emphasized reaction to flicker during single flicker cycles. We did not identify a plausible explanation for such a divergent venous response strategy. Pathophysiological alterations in blood pressure or blood gases cannot be excluded, as these were not part of the original study protocol. Presumably, a simultaneous qualitative assessment of both retinal arteries and veins would also help to elucidate the issue. On the other hand, tracking of vessel diameter for the same amount of the time without using flicker, maybe even with lower light intensity may provide useful information to better understand this variability.

After our first measurements in mice, it was discovered that repetitive 350 s DVA measurements with flickering light resulted in a reduction of retinal venous reaction. In 7 independent measurements, initial vasodilatation was abolished after repeating DVA twice (**S6 Fig)**. Venous responses varied during retinal recordings, yielding stronger responses during primary measurements, which may be partially a consequence of dilution of signaling molecules over time or the neuronal activity being attenuated by the progressive illumination, respectively. Moreover, repeated measurements revealed a reduction in reaction amplitude that may be partially related to the cumulative exposure to light.

Visual stimulation during the recording procedure may cause stress on the examined retina, as demonstrated postmortem when comparing flicker-exposed with flicker-naïve retinae (**Fig 4**). However, the examinations were not carried out simultaneously which can affect these results.

Using an infrared condition before and during flicker measurements could possibly be beneficial. Quite surprising is the observation that veins with smaller diameter showed a reduced response than veins with larger diameter (**S3 Fig**); this is in contrast to human findings [31]. Distance to ONH, however, did not influence venous reactivity. However, the shape of the flicker response seemed to be different for proximal and distal vessel area (**S3 Fig)**, possibly due to the different distribution of the cones in the mouse [32].

This first pilot study on the recording of murine retinal vessel responses aimed to report and investigate main peculiarities of retinal NVC in mice. Further optimization using fluorescent angiography, combining with infrared imaging, may render this model even more suitable for a variety of experimental setups.

## Conclusion

We present a novel non-contact imaging system to evaluate murine retinal vessel diameter and their response to flicker light (neurovascular coupling). While characteristic changes were readily observed in retinal veins, arterial investigation is currently hampered by technical limitations in a majority of animals, which may be overcome by use of contrast media (fluorescein/ICG). Retinal neuro-vascular capacity may exhaust during repetitive examination, suggesting the need for recovery time; due to the length of time between investigations, the role of adaptation/exhaustion vs the extent of permanent disruption, however, will have to be addressed in future projects. The imaging system holds the potential for basic research on vessel behavior and enables the investigation of microcirculation in wild-type and genetically modified animals.

## Supporting information

S1 Table Parameter of retinal venous reaction to flickering light: best vs. worst reaction per mouse. n = 21, age 4.7 (4.0–5.7) Mo.; median (1^st^ quartile– 3^rd^ quartile). Statistical comparison with Kruskal-Wallis test. Explorative testing without correction to multiple comparisons.
(DOCX)

S2 Table Parameter of retinal venous reaction to flickering light in cycles: Best reaction per mouse. n = 21. Statistical comparison with Kruskal-Wallis test.
(DOCX)

S3 Table Parameter of retinal venous reaction to flickering light: Proximal vs. distal vessels. n = 23 / 21; age: 4.6 (4.0–5.7) Mo. vs. 4.6 (4.0–5.4) Mo. (p = 0.961); median (1^st^ quartile– 3^rd^ quartile), significance with Mann-Whitney-U-test. Explorative testing without correction to multiple comparisons. (DOCX)

S4 Table Retinal venous responses for individual cases. (DOCX)

S1 Fig Schematic representation of neurovascular coupling (NVC) in murine retina. The vessel diameter is recorded continuously in an operator-selected region of interest. Standard 350 sec dynamic vessel analysis measurement protocol is used. The baseline (100%) is followed by flicker light stimulation (20 sec). Physiological response curve of retinal vessel features primary vasodilation after initiation of the flicker light impulse. Termination of the stimulus is typically followed by a reflexive vasoconstriction. AUC area under the curve.
(TIFF)

S2 Fig Average reaction of the best arterial reactions in this study, reaction in 8 mice (14 measurements). (TIFF)

S3 Fig **A.** Comparison of retinal venous response to flicker divided in small vessels and large vessels. Average reactions in subgroups n = 30. **B.** Comparison of retinal venous response to flicker according to the location of the measured segment on the fundus. Proximal vs. distal vessels (n = 23 / 21).
(TIFF)

S4 Fig **A.** comparison of the best and the worst retinal venous reaction in each mouse, n = 21. **B.** The averaged best reaction curve from the top panel is compared with retinal venous reactions in healthy. The averaged best reaction curve from the top panel is compared with retinal venous reactions in healthy young male (first author, W.A., the test measurement was performed prior to the study reported previously (Albanna et al. 2016). The same DVA protocol was used for both, mice and human. Monochromatic rectangular flash light impulses at 530 at a frequency of 12.5 Hz for 20 s each, alternating with 80 s of steady illumination.
(TIFF)

S5 Fig Examples of increasing and decreasing venous responses. **AI.** Individual reactions to flicker light during 3 cycles within one stimulation course. Vessel diameter decreases from 1^st^ to 3^rd^ flicker cycle, after each response (decrease). **BI.** Vessel diameter increases from 1^st^ to 3^rd^ flicker cycle, after each response (increase). **AII & BII**: Corresponding averaged responses with relative vessel diameter changes in % to individual baseline.
(TIFF)

S6 Fig Retinal venous responses in primary vs. repeated flicker experiment on the same mice at the same vessel segment. Average reactions in groups n = 7, time interval between the repeated measurements, 11.0 (9.0–12.5) min.
(TIFF)
