# Supplementary material for: Non-invasive evaluation of neurovascular coupling in the murine retina by dynamic retinal vessel analysis
Source: PLoS One. 2018 Oct 4;13(10):e0204689. doi: 10.1371/journal.pone.0204689 (PMC6171857; doi:10.1371/journal.pone.0204689)
Supplement: S1 Table — Statistical comparison with Kruskal-Wallis test. Explorative testing without correction to multiple comparisons. (DOCX) [file pone.0204689.s001.docx]

**S1 Table**

Parameter of retinal venous reaction to flickering light: best vs. worst reaction per mouse. n = 21, age 4.7 (4.0 – 5.7) Mo.; median (1^st^ quartile – 3^rd^ quartile). Statistical comparison with Kruskal-Wallis test. Explorative testing without correction to multiple comparisons.

| **parameter/group** | **best reaction** | **worse reaction** | **p - value** |
| --- | --- | --- | --- |
| data quality,  [subjective score 1.0 – 5.0 ] | 4.5(4.0 – 5.0) | 4.0 (3.8 – 4.5) | 0.354 |
| venous diameter [MU] | 58.0 (47.4 – 63.4) | 58.8 (47.1 –63.7) | 0.456 |
| mean maximal venous dilation [% baseline] | 1.4 (1.0 – 1.9) | 0.4 (0.2 – 1.1) | **<0.001** |
| time of maximal venous dilation [s] | 12.0 (9.0 – 20.0) | 24.0 (11.0 – 27.0) | 0.073 |
| venous dilation at the flicker cessation [% baseline] | 0.8 (0.3 – 1.5) | 0.3 (-0.7 – 0.2) | **<0.001** |
| venous reactive magnitude  [% baseline] | 2.2 (1.7 – 3.8) | 1.4 (1.3 – 2.0) | 0.009 |
| venous AUC during the flicker[%*s] | 11.6 (3.4 – 21.7) | 1.5 (-11.3 – 3.4) | **<0.001** |
| venous time of center of gravity at flicker[s] | 17.1 (11.3 – 33.9) | 39.8 (23.5 – 62.5) | 0.088 |
| mean maximal venous constriction [% baseline] | -1.1(-2.0 – -0.6) | -1.1(-1.8 – -0.6) | 0.481 |
