# Supplementary material for: Non-invasive evaluation of neurovascular coupling in the murine retina by dynamic retinal vessel analysis
Source: PLoS One. 2018 Oct 4;13(10):e0204689. doi: 10.1371/journal.pone.0204689 (PMC6171857; doi:10.1371/journal.pone.0204689)
Supplement: S2 Table — n = 21. Statistical comparison with Kruskal-Wallis test. (DOCX) [file pone.0204689.s002.docx]

**S2 Table**

Parameter of retinal venous reaction to flickering light in cycles: best reaction per mouse. n = 21. Statistical comparison with Kruskal-Wallis test.

| **parameter / flicker cycle** | **Cycle 1** | **Cycle 2** | **Cycle 3** | **p-value** |
| --- | --- | --- | --- | --- |
| baseline diameter, [MU] | 57.5 (48.9 – 63.6) | 57.7 (45.8 – 63.4) | 56.9 (45.7 – 62.9) | 0.966 |
| maximal venous dilation, [MU] | 60.8 (49.4 – 65.2) | 59.9 (48.9 – 65.0) | 58.4 (49.3 – 65.5) | 0.679 |
| mean maximal venous dilation, [MU] | 60.1 (49.2 – 64.2) | 59.0 (47.8 – 63.8) | 56.8 (48.6 – 64.2) | 0.184 |
| time of maximal venous dilation, [s] | 10.0 (6.0 – 15.0) | 15.0 (8.8 – 21.5) | 11.5 (9.0 – 18.3) | 0.967 |
| maximal venous dilation, relative to the baseline, [%] | 1.0 (0.2 – 2.0) | 1.3 (0.4 – 2.0) | 0.9 (0.4 – 2.8) | 0.304 |
