# Supplementary material for: Non-invasive evaluation of neurovascular coupling in the murine retina by dynamic retinal vessel analysis
Source: PLoS One. 2018 Oct 4;13(10):e0204689. doi: 10.1371/journal.pone.0204689 (PMC6171857; doi:10.1371/journal.pone.0204689)
Supplement: S3 Table — (DOCX) [file pone.0204689.s003.docx]

**S3 Table**

Parameter of retinal venous reaction to flickering light: proximal vs. distal vessels. n = 23 / 21; age: 4.6 (4.0 – 5.7) Mo. vs. 4.6 (4.0 – 5.4) Mo. (p= 0.961); median (1^st^ quartile – 3^rd^ quartile), significance with Mann-Whitney-U-test. Explorative testing without correction to multiple comparisons.

| **parameter/group** | **proximal vessel segments** | **distal vessel segments** | **p - value** |
| --- | --- | --- | --- |
| data quality,  [subjective score 1.0 – 5.0 ] | 4.0 (3.5 – 4.5) | 4.0 (4.0 – 4.5) | 0.874 |
| venous diameter [MU] | 55.4 (45.5 – 62.1) | 54.6 (46.4 – 61.7) | 0.842 |
| mean maximal venous dilation [% baseline] | 0.8 (0.4 – 1.4) | 1.1 (0.3 – 1.7) | 0.613 |
| time of maximal venous dilation [s] | 14.0 (9.0 – 19.5) | 18.0 (9.0 – 22.0) | 0.371 |
| venous dilation at the flicker cessation [% baseline] | 0.3 (-0.2 – 0.9) | 0.7 (-0.1 – 1.0) | 0.458 |
| venous reactive magnitude  [% baseline] | 1.8 (1.2 – 2.5) | 1.8 (1.7 – 3.7) | 0.495 |
| venous AUC during the flicker [%*s] | 5.3 (0.4 – 12.3) | 8.4 (1.2 – 18.4) | 0.690 |
| venous time of center of gravity at flicker [s] | 24.9 (11.3 – 44.2) | 15.9 (12.0 – 31.0) | 0.560 |
| mean maximal venous constriction [% baseline] | -1.0 (-1.5 – -0.5) | -0.9 (-2.1 – -0.4) | 0.629 |
