# Supplementary material for: Non-invasive evaluation of neurovascular coupling in the murine retina by dynamic retinal vessel analysis
Source: PLoS One. 2018 Oct 4;13(10):e0204689. doi: 10.1371/journal.pone.0204689 (PMC6171857; doi:10.1371/journal.pone.0204689)
Supplement: S4 Table — (DOCX) [file pone.0204689.s004.docx]

**S4 Table:** Retinal venous responses for individual cases.

| **Nr.** | **Mouse** | **data quality, arbitrary units** | **venous diameter, [MU]** | **mean maximal venous dilation, [% baseline]** | **venous dilation at the flicker cessation, [% baseline]** | **time of maximal venous dilation, [s]** | **venous AUC during the flicker, [%*s]** |
| --- | --- | --- | --- | --- | --- | --- | --- |
| 1 | Mouse 1 | 4.0 | 59.3 | 0.5 | -0.7 | 11.0 | 3.4 |
| 2 | Mouse 2 | 4.0 | 53.1 | 0.4 | -0.2 | 9.0 | 2.3 |
| 3 | Mouse 2 | 4.0 | 32.1 | -0.4 | -1.4 | 40.0 | -20.2 |
| 4 | Mouse 2 | 2.5 | 58.0 | 2.9 | 0.1 | 8.0 | 21.7 |
| 5 | Mouse 2 | 4.0 | 52.3 | 0.4 | -0.1 | 7.0 | 0.4 |
| 6 | Mouse 3 | 4.0 | 62.7 | 0.3 | -0.4 | 12.0 | -3.1 |
| 7 | Mouse 3 | 4.5 | 65.4 | -0.4 | -0.6 | 24.0 | -11.7 |
| 8 | Mouse 3 | 4.0 | 65.4 | 0.1 | -0.8 | 15.0 | -14.1 |
| 9 | Mouse 3 | 4.0 | 62.0 | 0.0 | -0.6 | 0.0 | -22.1 |
| 10 | Mouse 3 | 3.5 | 44.2 | 3.0 | 2.6 | 21.0 | 6.2 |
| 11 | Mouse 4 | 5.0 | 61.1 | 0.8 | 0.8 | 23.0 | 10.4 |
| 12 | Mouse 4 | 3.5 | 51.3 | 0.5 | 0.3 | 21.0 | -2.3 |
| 13 | Mouse 4 | 4.0 | 48.6 | 0.1 | 0.0 | 29.0 | -3.0 |
| 14 | Mouse 4 | 3.5 | 49.6 | 1.2 | 1.0 | 21.0 | 8.4 |
| 15 | Mouse 4 | 2.5 | 56.7 | 0.3 | -2.3 | 4.0 | -25.4 |
| 16 | Mouse 4 | 5.0 | 61.1 | 0.6 | 0.6 | 20.0 | 8.5 |
| 17 | Mouse 4 | 4.5 | 61.7 | 0.6 | 0.5 | 19.0 | 4.0 |
| 18 | Mouse 4 | 4.0 | 67.7 | 0.7 | 0.3 | 33.0 | 2.6 |
| 19 | Mouse 5 | 3.5 | 45.1 | 0.6 | -0.6 | 24.0 | -15.9 |
| 20 | Mouse 5 | 4.0 | 47.5 | 0.2 | -0.7 | 40.0 | -17.3 |
| 21 | Mouse 5 | 4.0 | 46.8 | 0.7 | -0.6 | 24.0 | -9.2 |
| 22 | Mouse 5 | 4.0 | 41.3 | 0.9 | 1.0 | 18.0 | 10.5 |
| 23 | Mouse 6 | 5.0 | 62.9 | 1.6 | 0.0 | 38.0 | -1.4 |
| 24 | Mouse 6 | 4.0 | 38.0 | 0.8 | 0.3 | 25.0 | 4.4 |
| 25 | Mouse 6 | 4.5 | 34.0 | 1.7 | 0.8 | 2.0 | 24.8 |
| 26 | Mouse 6 | 4.5 | 34.0 | 1.5 | 0.3 | 10.0 | 19.3 |
| 27 | Mouse 6 | 4.0 | 33.9 | 1.0 | 0.9 | 17.0 | 9.3 |
| 28 | Mouse 7 | 3.5 | 46.2 | 0.7 | 0.6 | 18.0 | 5.0 |
| 29 | Mouse 7 | 3.0 | 57.0 | 0.3 | -0.3 | 26.0 | -20.0 |
| 30 | Mouse 7 | 4.5 | 69.1 | 0.5 | 0.6 | 9.0 | 5.3 |
| 31 | Mouse 7 | 4.5 | 68.4 | 0.5 | 0.3 | 9.0 | 7.5 |
| 32 | Mouse 7 | 3.5 | 47.4 | 1.6 | 1.5 | 18.0 | 11.6 |
| 33 | Mouse 8 | 5.0 | 40.0 | -0.2 | -0.8 | 1.0 | -11.0 |
| 34 | Mouse 8 | 5.0 | 54.3 | 0.0 | -0.3 | 1.0 | -7.6 |
| 35 | Mouse 8 | 4.5 | 53.7 | -0.1 | -0.2 | 20.0 | -6.2 |
| 36 | Mouse 8 | 4.5 | 43.5 | -0.2 | -0.7 | 1.0 | -11.3 |
| 37 | Mouse 8 | 5.0 | 43.4 | -0.1 | -0.7 | 9.0 | -8.4 |
| 38 | Mouse 8 | 5.0 | 46.2 | -0.1 | -0.6 | 15.0 | -8.5 |
| 39 | Mouse 9 | 4.0 | 91.9 | 1.8 | 0.7 | 26.0 | 4.2 |
| 40 | Mouse 9 | 4.5 | 86.6 | 1.6 | 1.5 | 22.0 | 2.6 |
| 41 | Mouse 10 | 3.5 | 55.4 | 1.2 | 0.8 | 10.0 | 16.2 |
| 42 | Mouse 10 | 3.5 | 58.8 | 1.7 | 0.9 | 27.0 | 9.8 |
| 43 | Mouse 11 | 5.0 | 58.8 | -0.7 | -0.9 | 12.0 | -16.1 |
| 44 | Mouse 11 | 5.0 | 54.1 | -0.3 | -0.5 | 4.0 | -8.8 |
| 45 | Mouse 11 | 5.0 | 44.2 | -0.3 | -0.7 | 30.0 | -12.3 |
| 46 | Mouse 11 | 5.0 | 43.0 | 0.2 | -0.1 | 4.0 | 1.2 |
| 47 | Mouse 11 | 5.0 | 44.8 | 0.3 | -0.2 | 14.0 | 1.5 |
| 48 | Mouse 11 | 5.0 | 48.9 | 0.7 | -0.3 | 4.0 | 4.4 |
| 49 | Mouse 12 | 5.0 | 64.5 | 0.4 | 0.2 | 12.0 | 3.7 |
| 50 | Mouse 12 | 4.5 | 42.6 | 0.4 | 0.2 | 25.0 | -0.4 |
| 51 | Mouse 12 | 4.0 | 68.2 | 0.9 | 0.8 | 20.0 | 2.2 |
| 52 | Mouse 13 | 5.0 | 54.6 | 0.7 | 0.7 | 19.0 | 6.9 |
| 53 | Mouse 13 | 5.0 | 47.1 | -0.2 | -0.6 | 16.0 | -9.4 |
| 54 | Mouse 13 | 4.5 | 45.0 | 0.2 | -0.4 | 12.0 | -1.0 |
| 55 | Mouse 13 | 4.5 | 57.4 | 0.8 | -0.2 | 13.0 | 10.0 |
| 56 | Mouse 13 | 4.5 | 48.8 | 1.1 | 0.3 | 7.0 | 13.9 |
| 57 | Mouse 13 | 5.0 | 59.4 | 1.1 | 0.7 | 27.0 | 9.9 |
| 58 | Mouse 14 | 5.0 | 36.8 | 2.2 | 1.3 | 12.0 | 29.9 |
| 59 | Mouse 14 | 4.0 | 35.1 | 0.0 | -0.2 | 12.0 | -3.5 |
| 60 | Mouse 14 | 3.0 | 37.1 | 1.8 | 1.1 | 23.0 | 14.0 |
| 61 | Mouse 14 | 4.0 | 33.6 | 0.8 | 0.2 | 28.0 | 4.2 |
| 62 | Mouse 14 | 5.0 | 37.5 | 1.0 | 0.6 | 24.0 | 11.5 |
| 63 | Mouse 15 | 5.0 | 65.5 | 1.0 | 0.5 | 29.0 | 9.7 |
| 64 | Mouse 15 | 4.0 | 65.6 | 1.6 | 0.7 | 27.0 | 3.1 |
| 65 | Mouse 16 | 4.5 | 46.4 | 0.9 | 0.7 | 2.0 | 15.6 |
| 66 | Mouse 16 | 3.5 | 55.4 | 1.8 | 0.2 | 9.0 | 29.2 |
| 67 | Mouse 16 | 3.5 | 53.9 | 1.3 | -0.7 | 25.0 | -1.5 |
| 68 | Mouse 16 | 3.5 | 47.0 | 1.1 | -0.4 | 37.0 | -4.0 |
| 69 | Mouse 16 | 2.5 | 49.7 | 3.8 | 0.6 | 14.0 | 12.0 |
| 70 | Mouse 16 | 3.5 | 60.2 | 4.5 | 3.9 | 21.0 | 42.7 |
| 71 | Mouse 17 | 5.0 | 63.4 | 1.9 | 1.5 | 10.0 | 33.0 |
| 72 | Mouse 17 | 5.0 | 65.5 | 1.0 | 0.0 | 12.0 | 9.2 |
| 73 | Mouse 17 | 2.5 | 51.2 | 2.1 | 1.7 | 40.0 | 31.6 |
| 74 | Mouse 18 | 5.0 | 61.8 | 4.5 | 3.0 | 23.0 | 43.0 |
| 75 | Mouse 18 | 3.5 | 49.4 | 1.2 | 0.4 | 14.0 | 11.4 |
| 76 | Mouse 18 | 4.0 | 63.7 | 0.9 | 0.7 | 16.0 | 14.4 |
| 77 | Mouse 18 | 4.0 | 63.7 | 0.2 | 0.0 | 9.0 | 2.0 |
| 78 | Mouse 19 | 5.0 | 63.9 | 0.3 | 0.1 | 17.0 | 0.5 |
| 79 | Mouse 19 | 3.5 | 65.8 | 0.1 | -0.3 | 11.0 | -4.3 |
| 80 | Mouse 20 | 5.0 | 64.3 | 0.3 | 0.1 | 28.0 | -4.7 |
| 81 | Mouse 20 | 4.0 | 52.4 | 0.2 | -0.7 | 2.0 | -5.7 |
| 82 | Mouse 20 | 4.5 | 60.4 | 1.4 | 0.8 | 9.0 | 20.9 |
| 83 | Mouse 20 | 5.0 | 59.0 | 0.5 | 0.0 | 31.0 | -0.1 |
| 84 | Mouse 20 | 3.5 | 58.1 | -0.2 | -0.2 | 18.0 | -5.1 |
| 85 | Mouse 21 | 3.5 | 60.9 | 2.6 | 1.8 | 7.0 | 42.3 |
| 86 | Mouse 21 | 4.0 | 58.6 | 1.1 | 0.9 | 16.0 | 17.8 |
| 87 | Mouse 21 | 3.5 | 51.0 | 1.5 | 0.9 | 29.0 | 10.5 |
| 88 | Mouse 21 | 4.0 | 52.6 | 1.1 | 0.4 | 35.0 | 5.8 |
| 89 | Mouse 21 | 4.0 | 57.4 | 1.2 | 1.2 | 18.0 | 18.4 |
| 90 | Mouse 21 | 3.5 | 59.9 | 0.7 | 0.0 | 7.0 | 8.7 |
