# Supplementary figures and images for: Non-invasive evaluation of neurovascular coupling in the murine retina by dynamic retinal vessel analysis
Source: PLoS One. 2018 Oct 4;13(10):e0204689. doi: 10.1371/journal.pone.0204689 (PMC6171857; doi:10.1371/journal.pone.0204689)

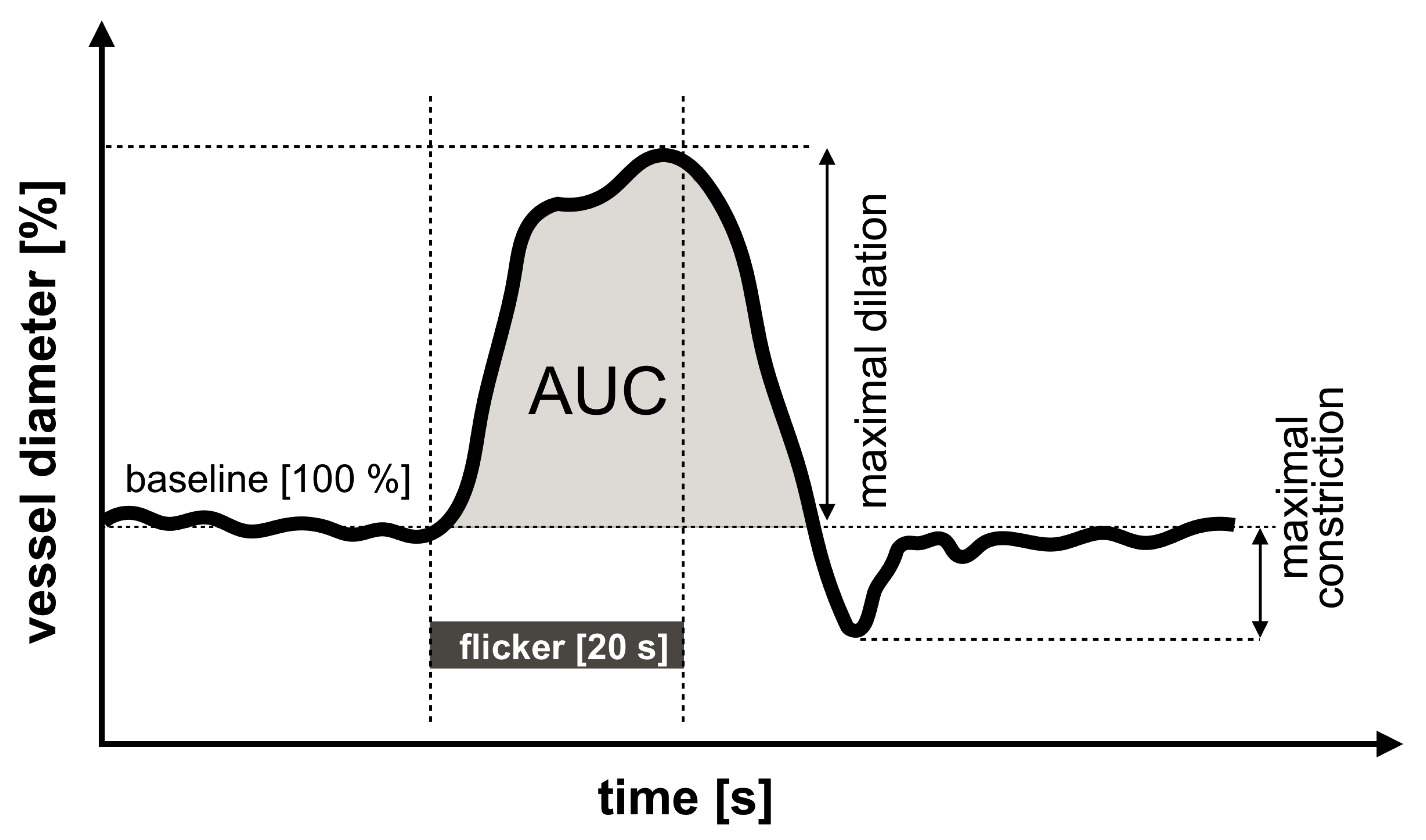

Supplement: S1 Fig — The vessel diameter is recorded continuously in an operator-selected region of interest. Standard 350 sec dynamic vessel analysis measurement protocol is used. The baseline (100%) is followed by flicker light stimulation (20 sec). Physiological response curve of retinal vessel features primary vasodilation after initiation of the flicker light impulse. Termination of the stimulus is typically followed by a reflexive vasoconstriction. AUC area under the curve. (TIFF) [file pone.0204689.s005.tiff]

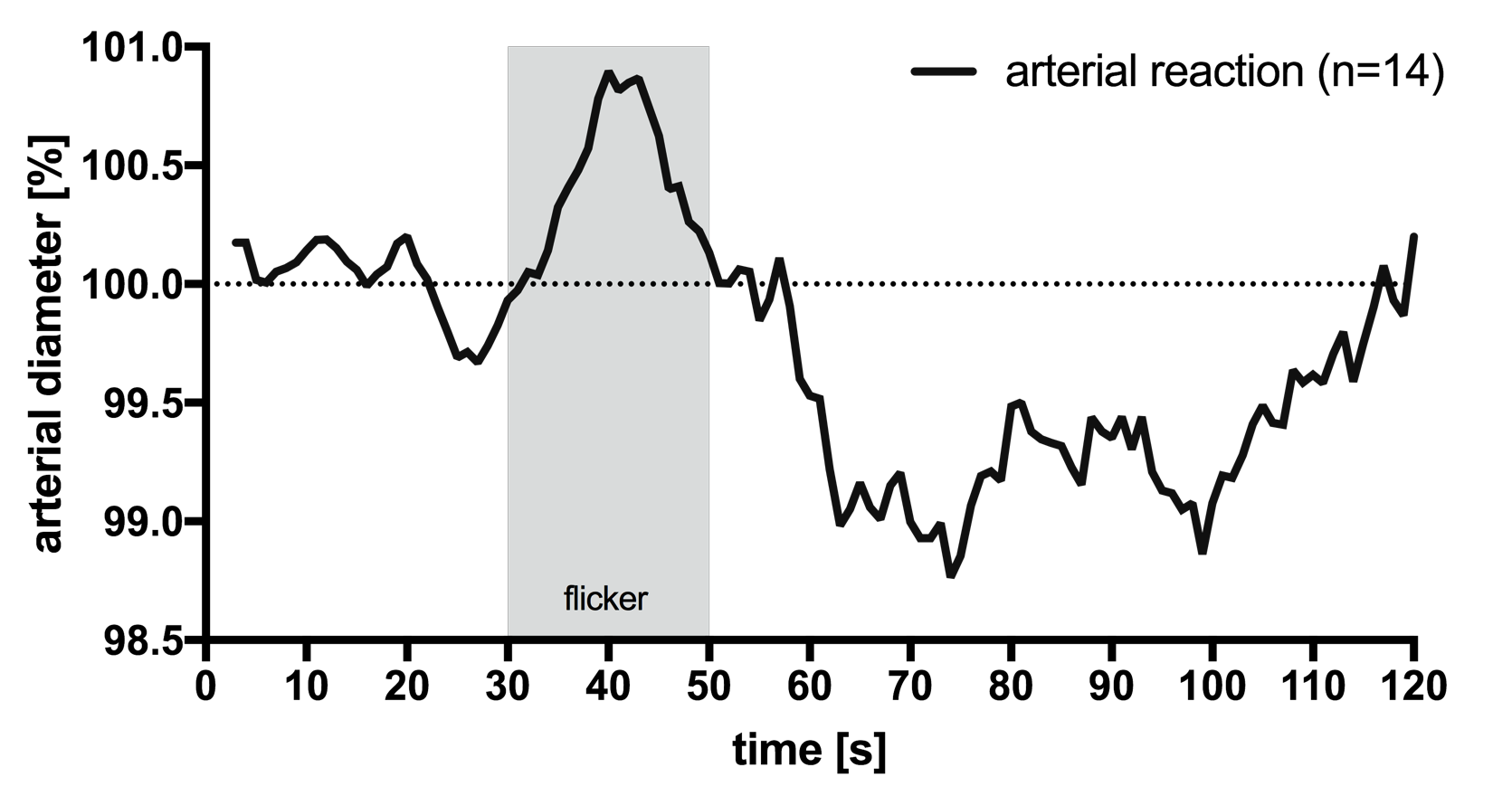

Supplement: S2 Fig — (TIFF) [file pone.0204689.s006.tiff]

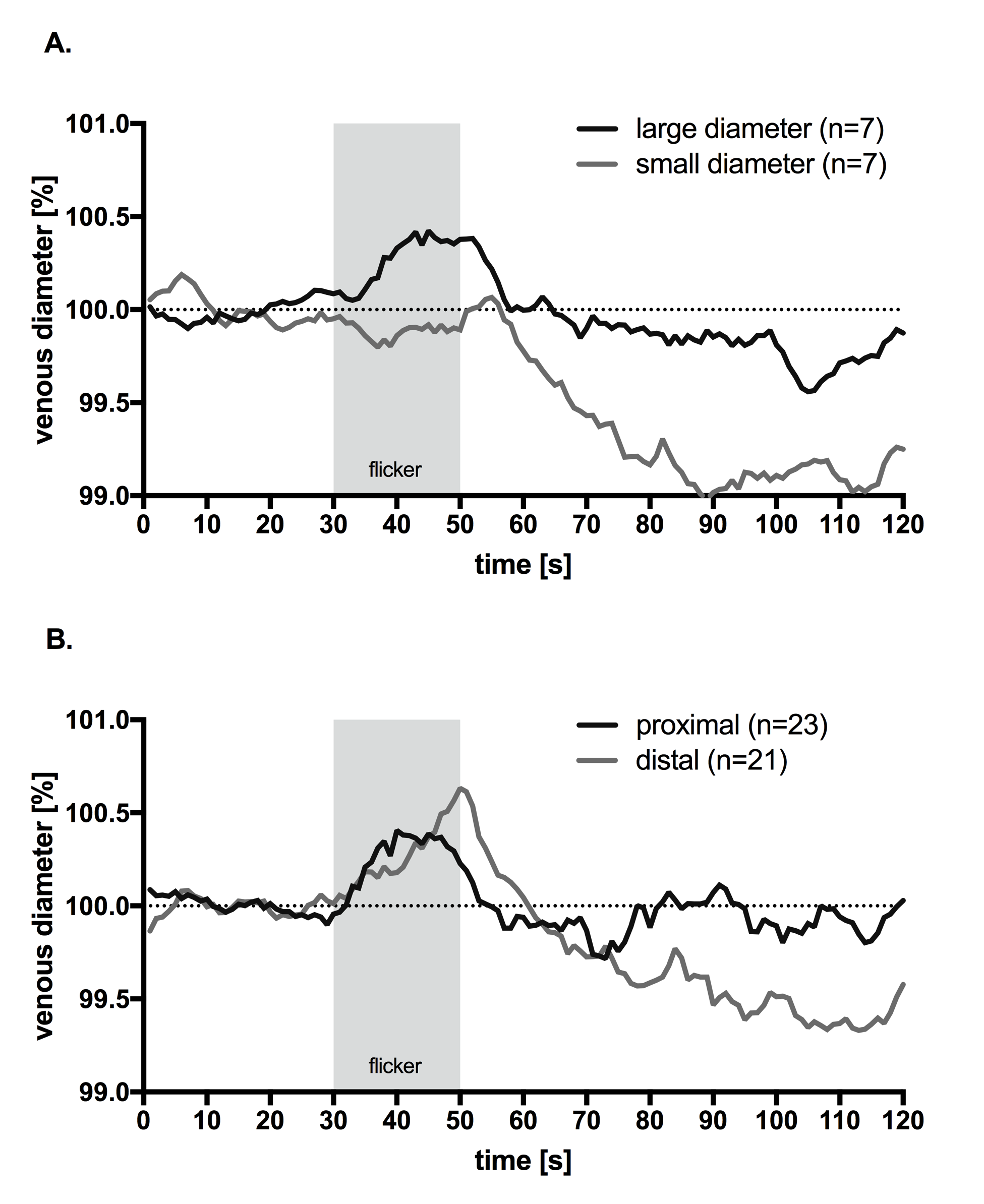

Supplement: S3 Fig — A. Comparison of retinal venous response to flicker divided in small vessels and large vessels. Average reactions in subgroups n = 30. B. Comparison of retinal venous response to flicker according to the location of the measured segment on the fundus. Proximal vs. distal vessels (n = 23 / 21). (TIFF) [file pone.0204689.s007.tiff]

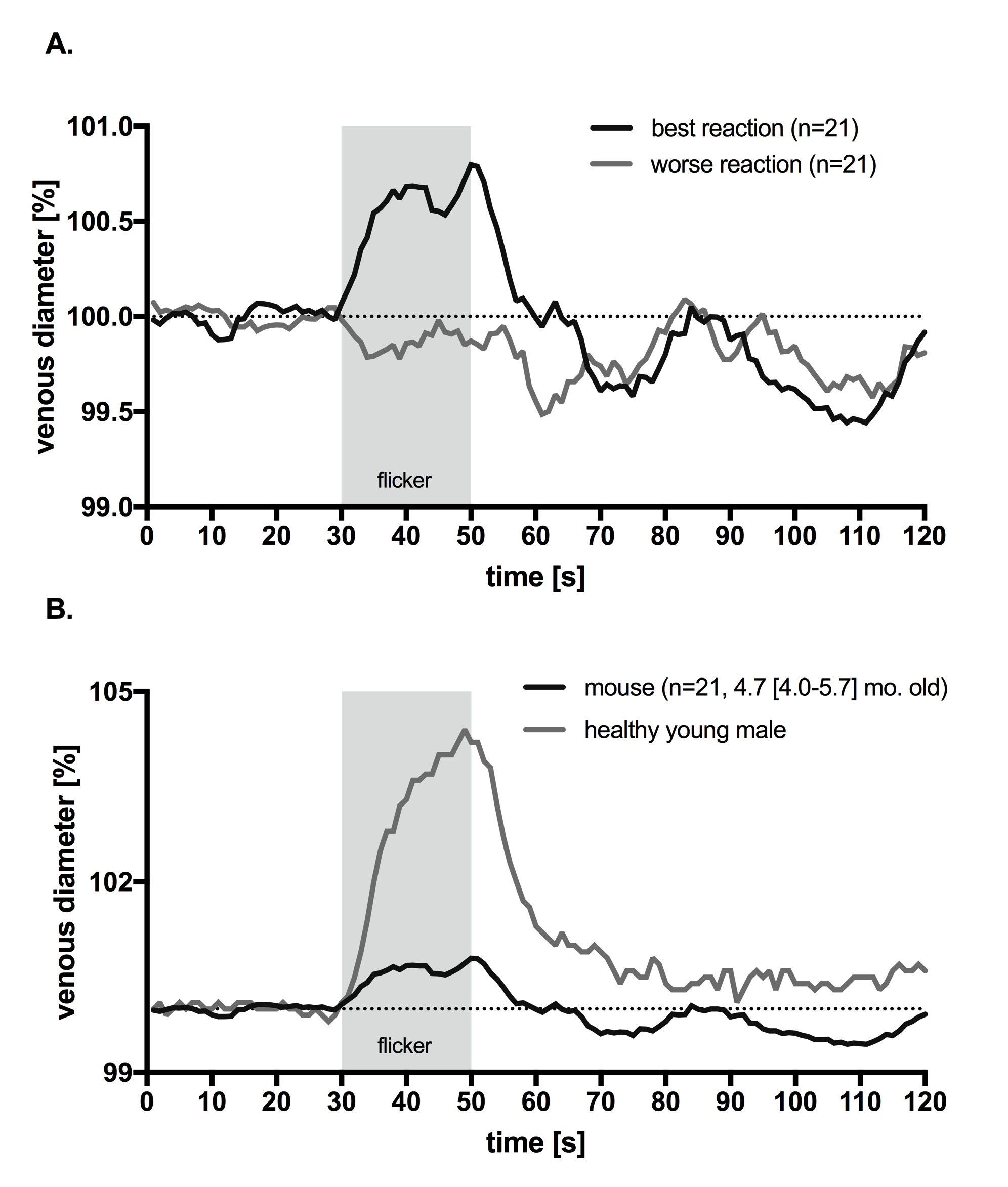

Supplement: S4 Fig — A. comparison of the best and the worst retinal venous reaction in each mouse, n = 21. B. The averaged best reaction curve from the top panel is compared with retinal venous reactions in healthy. The averaged best reaction curve from the top panel is compared with retinal venous reactions in healthy young male (first author, W.A., the test measurement was performed prior to the study reported previously (Albanna et al. 2016). The same DVA protocol was used for both, mice and human. Monochromatic rectangular flash light impulses at 530 at a frequency of 12.5 Hz for 20 s each, alternating with 80 s of steady illumination. (TIFF) [file pone.0204689.s008.tiff]

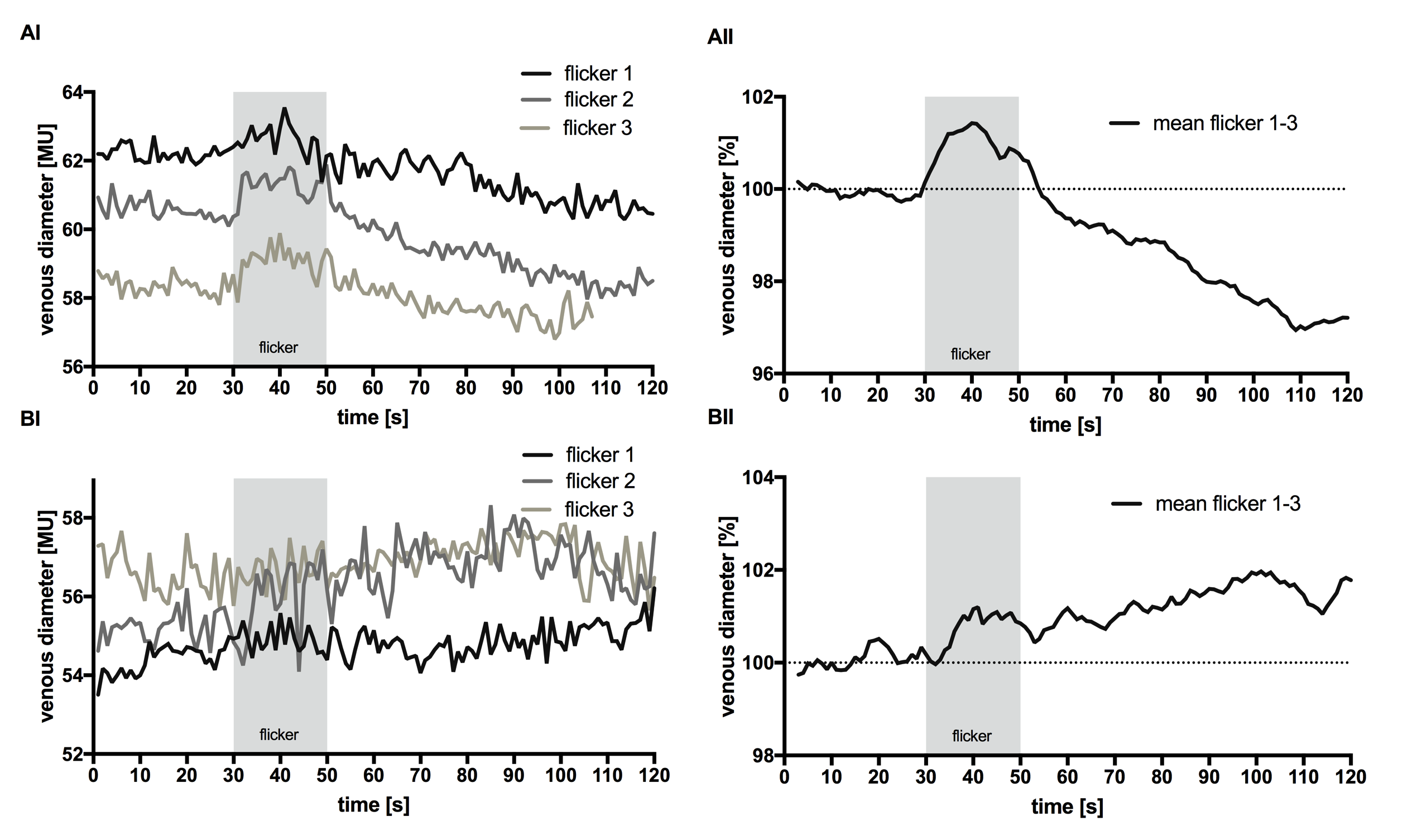

Supplement: S5 Fig — AI. Individual reactions to flicker light during 3 cycles within one stimulation course. Vessel diameter decreases from 1st to 3rd flicker cycle, after each response (decrease). BI. Vessel diameter increases from 1st to 3rd flicker cycle, after each response (increase). AII & BII: Corresponding averaged responses with relative vessel diameter changes in % to individual baseline. (TIFF) [file pone.0204689.s009.tiff]

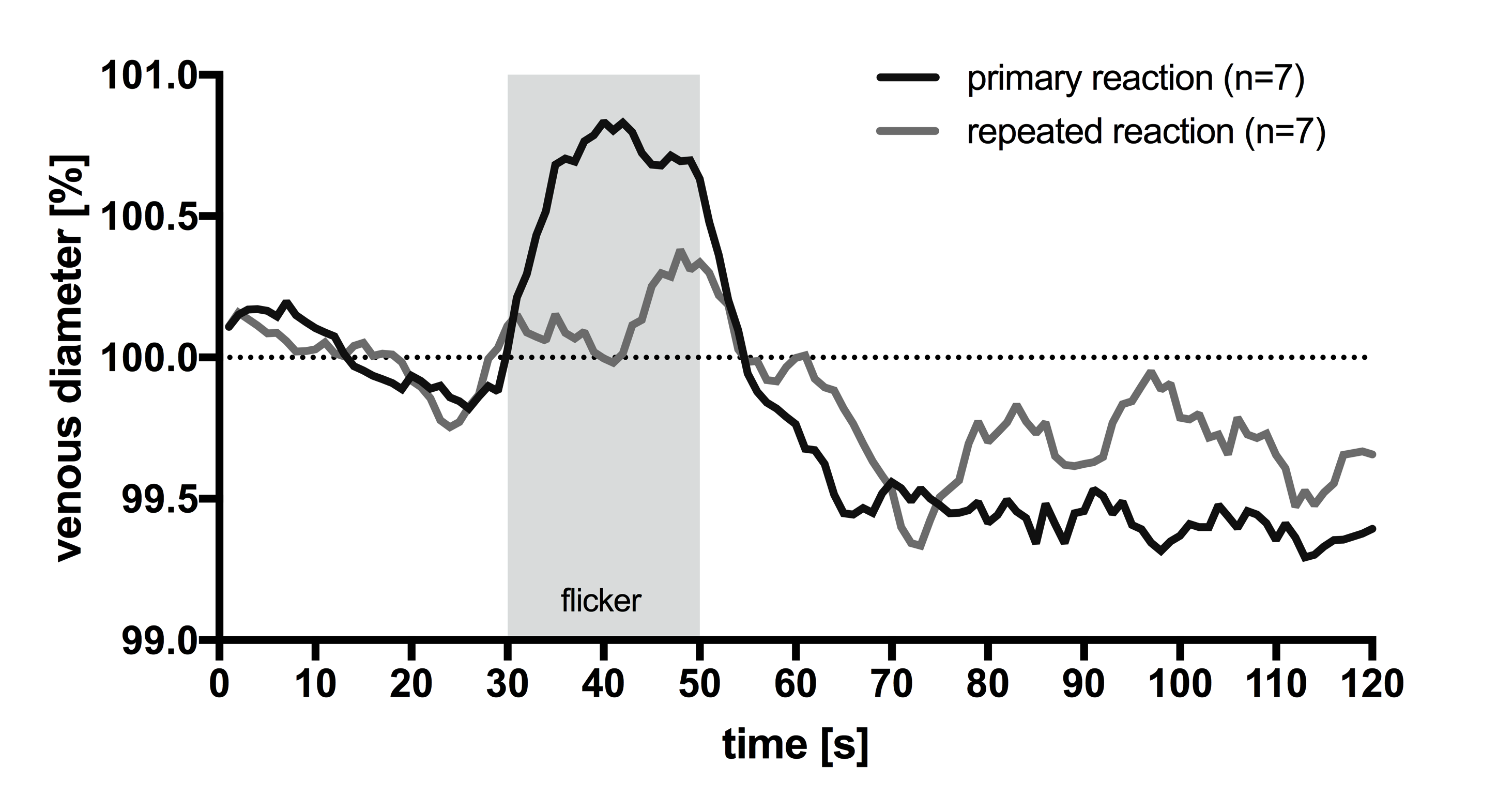

Supplement: S6 Fig — Average reactions in groups n = 7, time interval between the repeated measurements, 11.0 (9.0–12.5) min. (TIFF) [file pone.0204689.s010.tiff]
